# Supplementary material for: Changes in attitudes to vaccination as a result of the COVID-19 pandemic: A longitudinal study of older adults in the UK
Source: PLoS One. 2021 Dec 23;16(12):e0261844. doi: 10.1371/journal.pone.0261844 (PMC8699689; doi:10.1371/journal.pone.0261844)
Supplement: S1 File — (DOCX) [file pone.0261844.s001.docx]

**Supporting Information- File 1.**

**Here we provide the survey questions relevant for the data discussed in the manuscript. In addition to these questions, we also used the standardized 5C [16] and VAX [25] measures.**

Have you been vaccinated against Covid-19?

- Yes, I have had my first dose
- Yes, I have had both doses
- No, I declined the vaccination
- No, I have not been offered the vaccination
- Don't know

In the previous 12 months, did you receive the annual flu vaccination?

- Yes
- No
- Don't know

Please rank in order of importance the factors that influenced your decision to receive a Covid-19 vaccine, with 1 representing the most important factor.

______ To protect myself

______ To protect my family and friends

______ To help achieve community protection/ herd immunity

______ To bring an end to the restrictions

______ Other:

| 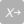 |
| --- |

Did you have any of the following concerns about receiving a Covid-19 vaccine? Please select all that apply.

- Worry about side-effects
- Worry that the speed of the clinical trials has been too fast
- Worry that it isn't safe
- Concern that the vaccine won't be effective
- I didn't have any concerns
- Other: ________________________________________________

To what extent did you find it difficult to decide whether or not to take the Covid-19 vaccine?

- Not at all difficult
- Somewhat difficult
- Very difficult
- Extremely difficult

|  |
| --- |

Thinking ahead to the next year.
Do you intend to accept the annual flu vaccine?

- Yes
- No
- Not sure

If you were offered further vaccinations against Covid-19 would you accept them?

- Yes
- No
- Not sure

We are interested in how you currently feel about vaccinations.

The standardized 5C [16] and VAX [25] scales followed.
