# Supplementary material for: Changes in attitudes to vaccination as a result of the COVID-19 pandemic: A longitudinal study of older adults in the UK
Source: PLoS One. 2021 Dec 23;16(12):e0261844. doi: 10.1371/journal.pone.0261844 (PMC8699689; doi:10.1371/journal.pone.0261844)
Supplement: Attachment — Submitted filename: Response to Reviewers.docx [file pone.0261844.s002.docx]

| **Reviewer Comments** | **Author Response** |
| --- | --- |
| **Journal Requirements** | |
| Journal Requirements:  When submitting your revision, we need you to address these additional requirements.  1. Please ensure that your manuscript meets PLOS ONE's style requirements, including those for file naming. The PLOS ONE style templates can be found at  https://journals.plos.org/plosone/s/file?id=wjVg/PLOSOne_formatting_sample_main_body.pdf and  https://journals.plos.org/plosone/s/file?id=ba62/PLOSOne_formatting_sample_title_authors_affiliations.pdf | The title page and manuscript have been revised to align with the *PLOS ONE* requirements. |
| 2. Thank you for stating the following in the Acknowledgments Section of your manuscript:  [The study was funded by the Chief Scientist Office, Scottish Government (Ref: CGA/19/52).] We note that you have provided funding information that is currently declared in your Funding Statement. However, funding information should not appear in the Acknowledgments section or other areas of your manuscript. We will only publish funding information present in the Funding Statement section of the online submission form.  Please remove any funding-related text from the manuscript and let us know how you would like to update your Funding Statement. Currently, your Funding Statement reads as follows:   [The study was funded by the Chief Scientist Office https://www.cso.scot.nhs.uk/, Scottish Government (Ref: Ref: CGA/19/52) research grant awarded to LW, LBN, SR, NC. The funders had no role in study design, data collection and analysis, decision to publish, or preparation of the manuscript.]  Please include your amended statements within your cover letter; we will change the online submission form on your behalf. | We have removed the funding related text from the acknowledgements section.  The Funding statement should read as “The study was funded by the Chief Scientist Office https://www.cso.scot.nhs.uk/, Scottish Government (Ref: Ref: CGA/19/52) research grant awarded to LW, LBN, SR, NC. The funders had no role in study design, data collection and analysis, decision to publish, or preparation of the manuscript” |
| 3. Have the authors made all data underlying the findings in their manuscript fully available?  The [PLOS Data policy](https://eur02.safelinks.protection.outlook.com/?url=http%3A%2F%2Fwww.plosone.org%2Fstatic%2Fpolicies.action%23sharing&data=04%7C01%7Clynn.williams%40strath.ac.uk%7C01fd8526c43f46096b6f08d9506ab629%7C631e0763153347eba5cd0457bee5944e%7C0%7C0%7C637629242683530495%7CUnknown%7CTWFpbGZsb3d8eyJWIjoiMC4wLjAwMDAiLCJQIjoiV2luMzIiLCJBTiI6Ik1haWwiLCJXVCI6Mn0%3D%7C1000&sdata=WUh09qqETdKtaXvq8tig5UBaZVatqG77Osmt8UmR7Rc%3D&reserved=0) requires authors to make all data underlying the findings described in their manuscript fully available without restriction, with rare exception (please refer to the Data Availability Statement in the manuscript PDF file). The data should be provided as part of the manuscript or its supporting information, or deposited to a public repository. For example, in addition to summary statistics, the data points behind means, medians and variance measures should be available. If there are restrictions on publicly sharing data—e.g. participant privacy or use of data from a third party—those must be specified | The dataset underlying this study is openly available and posted on Open Science Framework (https://doi.org/10.17605/OSF.IO/JYPN9). The link is included in the manuscript on lines 411-413 |
| **Reviewer 1** | |
| -The authors can benefit from additional relevant references in relation to Covid-19 vaccine hesitancy and its scope, flu and pneumococcal vaccine uptake among the elderly, among others:  E.g. Betsch, C., Rossmann, C., Pletz, M.W. et al. Increasing influenza and pneumococcal vaccine uptake in the elderly: study protocol for the multi-methods prospective intervention study Vaccination60+. BMC Public Health 18, 885 (2018). <https://doi.org/10.1186/s12889-018-5787-9>  Schmid P, Rauber D, Betsch C, Lidolt G, Denker ML (2017) Barriers of Influenza Vaccination Intention and Behavior – A Systematic Review of Influenza Vaccine Hesitancy, 2005 – 2016. PLOS ONE 12(1): e0170550. <https://doi.org/10.1371/journal.pone.0170550>  Fridman A, Gershon R, Gneezy A (2021) COVID-19 and vaccine hesitancy: A longitudinal study. PLOS ONE 16(4): e0250123. https://doi.org/10.1371/journal.pone.0250123 | We have included additional discussion in the introduction which references these papers (lines 97-99, 110-113, 117-123, 129-130) |
| The authors could provide more details regarding the recruitment process (e.g. which social media platforms were used) | We have added Facebook and Twitter to clarify which social media platforms were used in the recruitment process, as well as that the participant panels were recruited via email (see line 157) |
| The authors can benefit from mentioning the potential limitation involving sampling error that might have caused a biased estimation vaccine acceptance. | Thank you for this suggestion. We have added additional details in the limitations section to highlight the potential bias our online recruitment methods and online survey may have caused. Please see lines 365-369. |
| Did the authors check for internal consistency of the 5C and VAX scales? If so, can they provide the Cronbach alpha values? | We have added Cronbach’s 𝛂 for each subscale at both time points. Please see Table 2 (page13) for revisions. |
| Can the authors provide a decision or reference number of the ethical approval of the study by the School of Psychological Sciences & Health Ethics Committee at the University of Strathclyde? | The ethics statement entered in the submission system which would be published with the paper contains the reference number as follows “The study was approved by the School of Psychological Sciences & Health, University of Strathclyde ethics committee (Ref: 34/26/11/2019/Staff Williams). Participants provided written consent.” |
| -The authors are advised to submit the original questionnaire used in this study as a supplementary file to aid in the evaluation of their survey. | We have now included the survey as a supplementary file (referenced in line 210 in the manuscript). |
| **Reviewer 2** | |
| Reviewer #2: The paper is clear and it addresses a relevant topic. Nevertheless some major revisions are required. In particular  Introduction Please provide a brief description of the main tools used to investigate vaccine hesitancy (For example, see doi:10.15167/2421-4248/jpmh2020.61.3.1448) | We have now provided some information and a supporting reference about vaccine hesitancy measures. Please see lines 115-118 |
| Methods - Recruiting partecipants through social media posts and using an online questionnaire could lead selection bias, especially if the study aims to investigate older adults' attitudes. Please discuss this issue. | Thank you for this suggestion. We have added additional details in the limitations section to highlight the potential bias our online recruitment methods and survey may have caused. Please see lines 365-369. |
| Results Table 1. What "M" means? Plaese clarify Table 2. Please provide standard deviation/intrqurtile range | We have clarified in Table 1 that M represents Mean with a note of the abbreviations listed under the table (see page 11).  For Table 2, we have now included the Standard Deviation (see pages 12-13). We have also added the definitions for the abbreviations under this table as well. |
| Discussion There are some issues that could be adressed. In particular, the role of media and institutional comunication in determing attitudes towards vaccination should be described.  Additionally it could be interesting discussing on the role of the public debate on vaccination policies. For example, possible restrictions (such travel ban) can determine intetion to get vaccinated? | We have included additional details in the discussion from an evidence synthesis regarding media and COVID vaccination uptake (see lines 321-327). We have also provided additional context that the vaccine uptake rates may have been affected by broader social, cultural and political factors (please see lines 366-369) |
